# Supplementary material for: Comparative Analysis of Rhizosphere and Endosphere Fungal Communities in Healthy and Diseased Faba Bean Plants
Source: J Fungi (Basel). 2024 Jan 22;10(1):84. doi: 10.3390/jof10010084 (PMC10817651; doi:10.3390/jof10010084)
Supplement: Supplementary file 1 [file jof-10-00084-s001.zip › jof-2758058-supplementary.pdf]

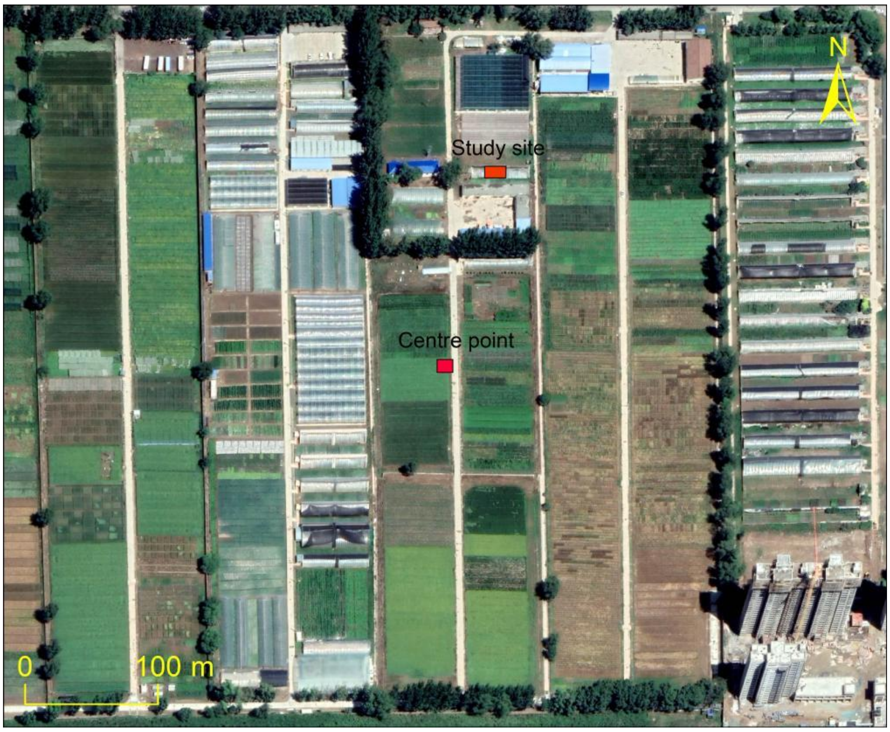

Figure S1. The map showing the location of the study site

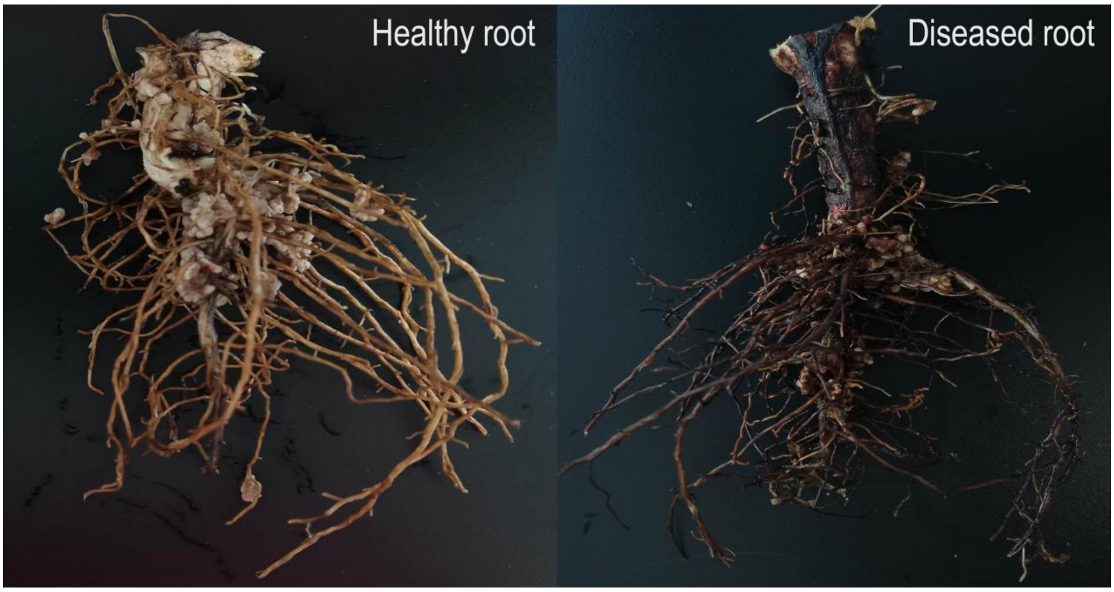

Figure S2. The picture showed the healthy and diseased roots

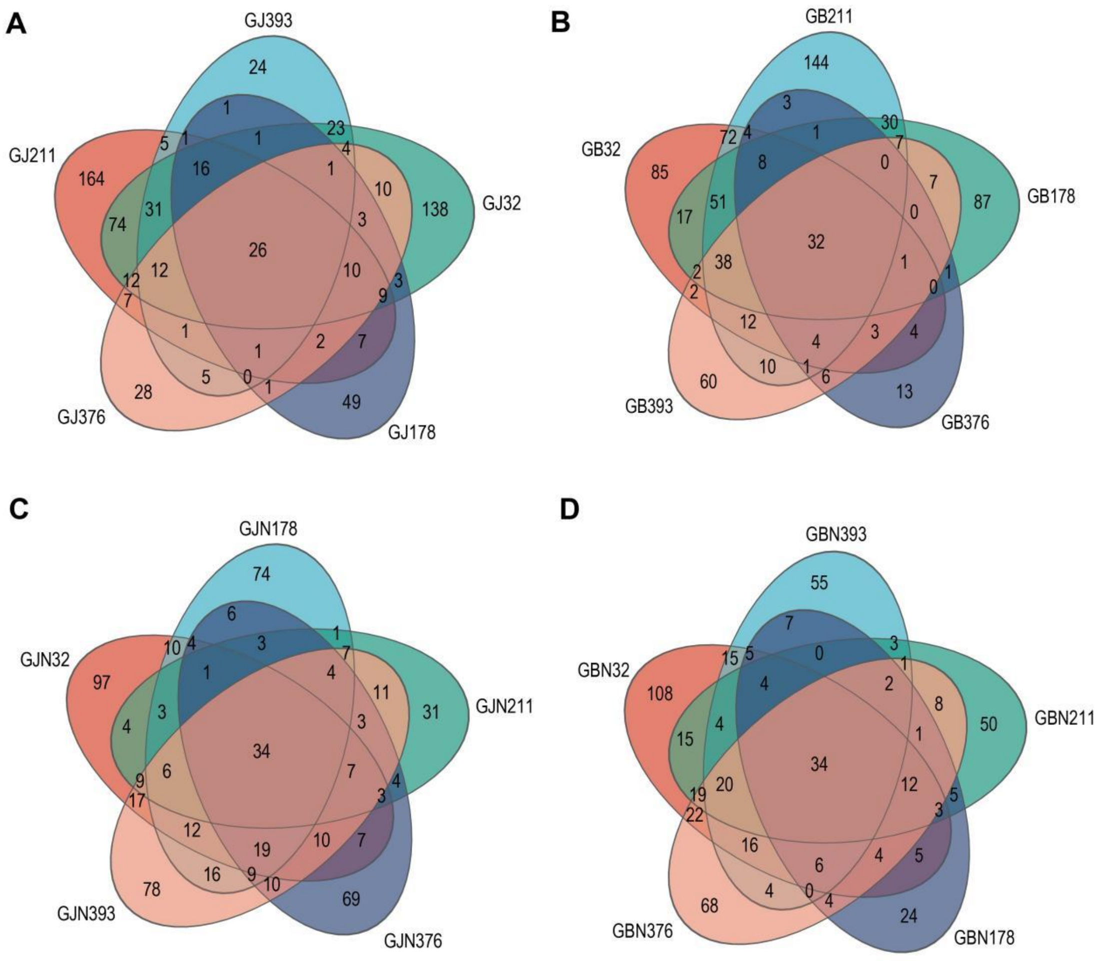

Figure S3. Venn diagram showing the numbers of fungal OTUs identified of healthy (A) and diseased (B) samples in rhizosphere soil and healthy (C) and diseased (D) samples in endosphere roots from different germplasms resources of faba bean.

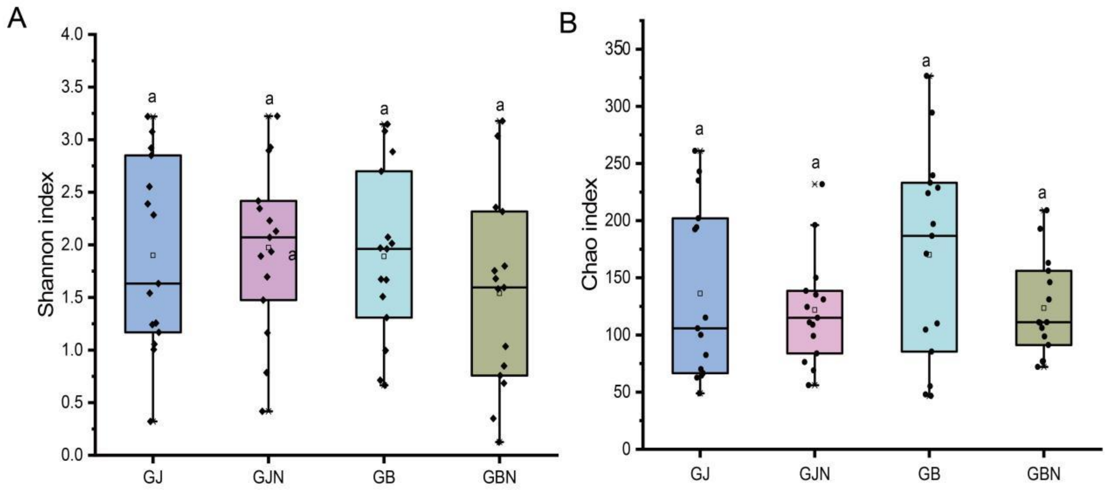

Figure S4. Alpha diversity indices of different groups. (A)Shannon index; (B)Chao index

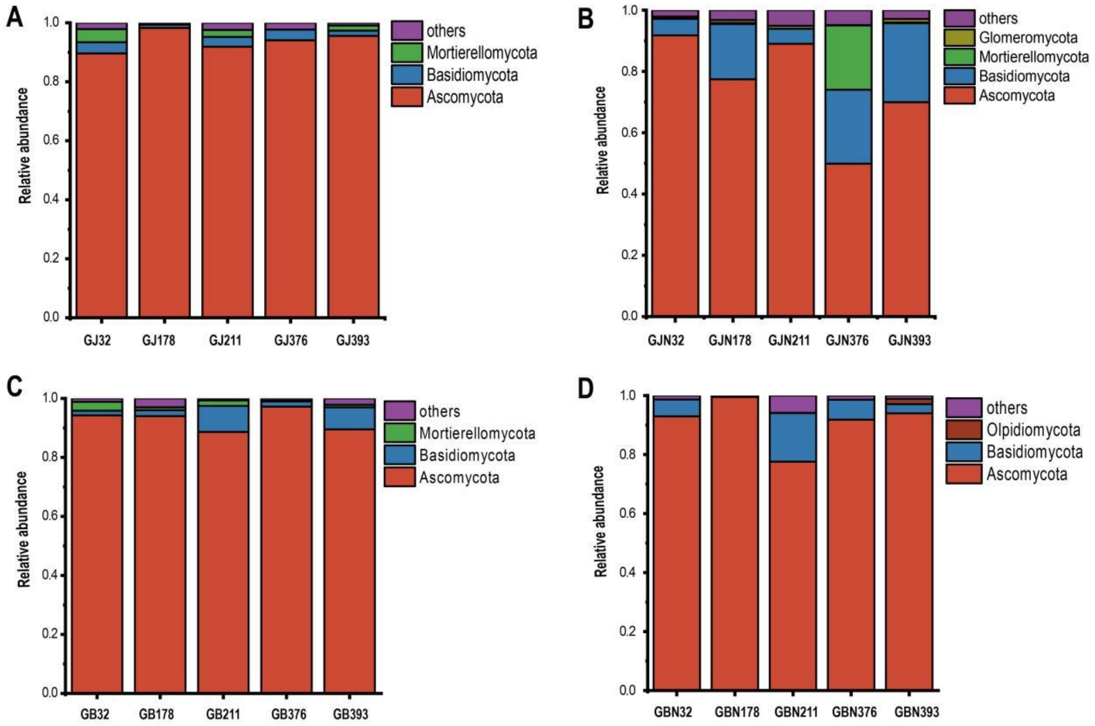

Figure S5. Fungal community composition of healthy (A) and diseased (C) samples in rhizosphere soil and of healthy (B) and diseased (D) samples in endosphere roots from different germplasms at the phylum level. The relative abundance making up less than 0.01%, and unclassified phyla were classified as "others."

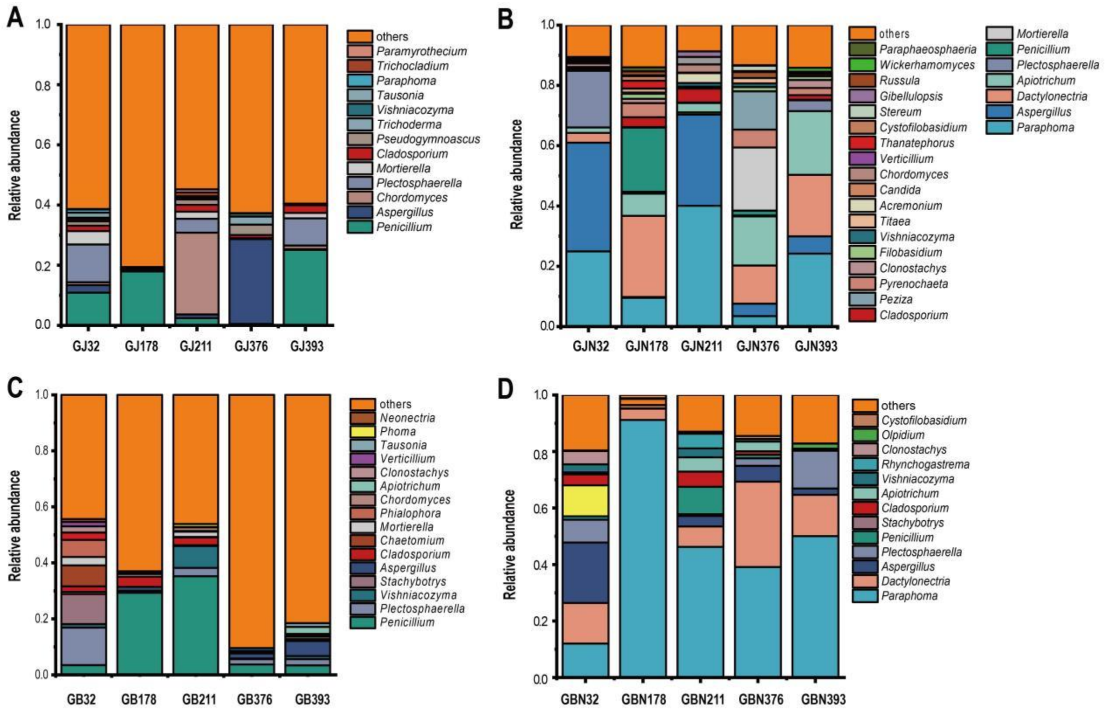

Figure S6. Fungal community composition of healthy (A) and diseased (C) samples in rhizosphere soil and of healthy (B) and diseased (D) samples in endosphere roots from different germplasms at the genus level. The relative abundance making up less than 0.01%, and unclassified genera were classified as “others.”

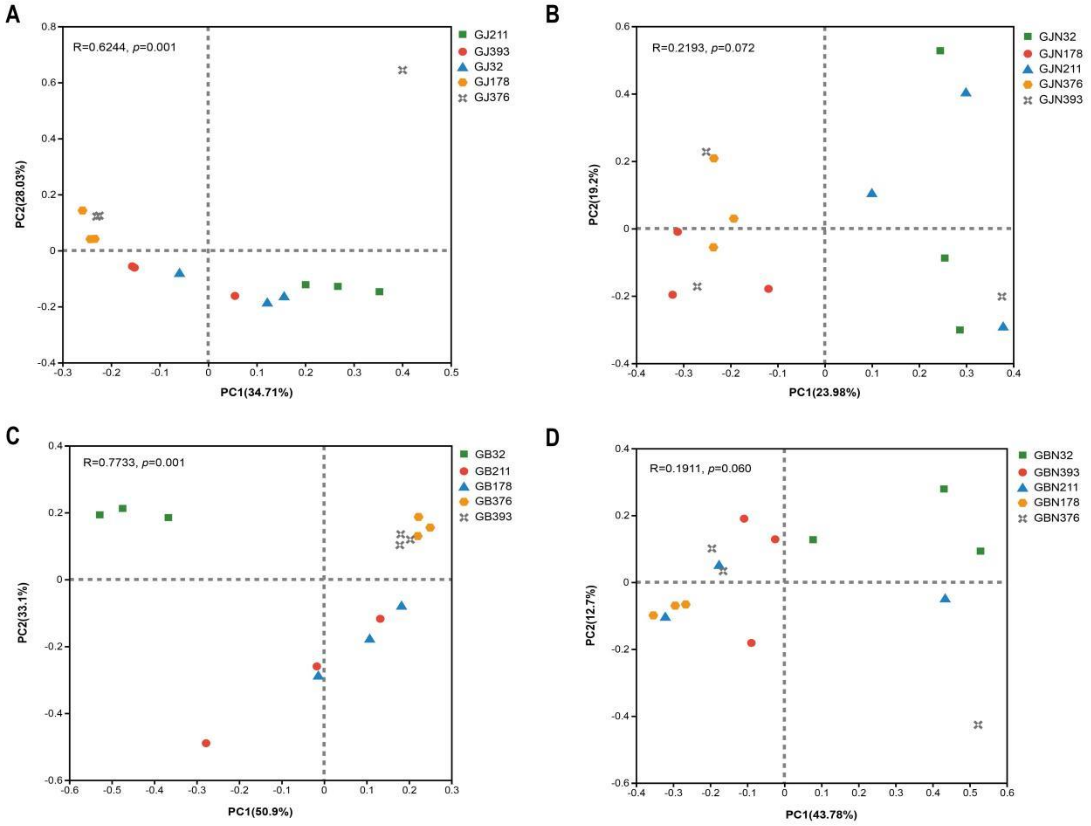

Figure S7. Principal Co-ordinate Analysis (PCoA) of fungal communities of healthy rhizosphere group (A), healthy endosphere group (B), diseased rhizosphere group (C), and diseased endosphere group (D) of different germplasms samples based on Bray-Curtis distances of the OTU matrix. 32, 211, 178, 376, 393 represent different germplasms of faba bean. GJ, GJN, GB and GBN represent healthy soil, healthy root, diseased soil and diseased root samples, respectively.

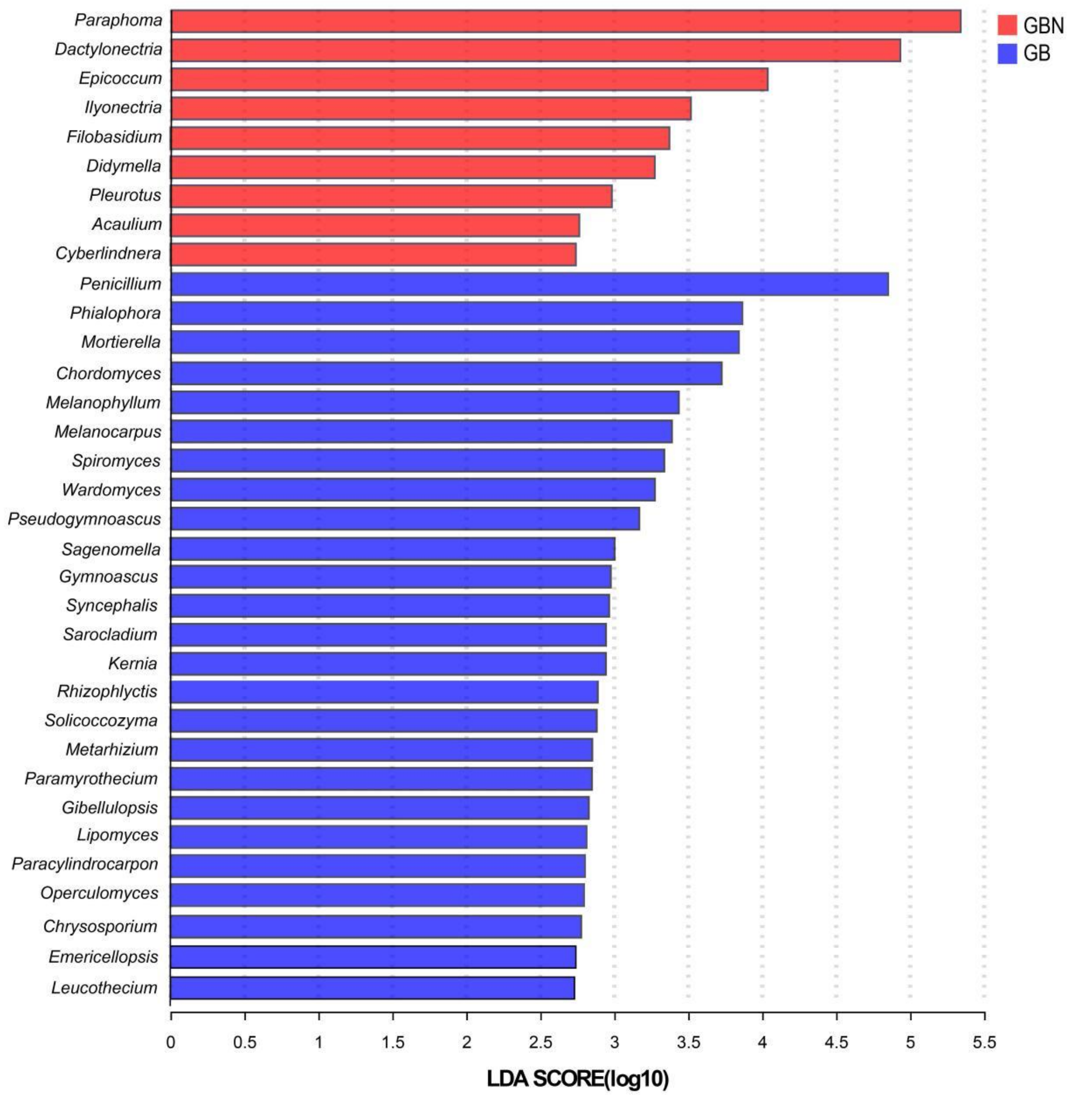

Figure S8. The LEfSe analysis the significantly enriched fungal community between GBN group and GB group. Fungal genera with the LDA score of more than 2 are shown.

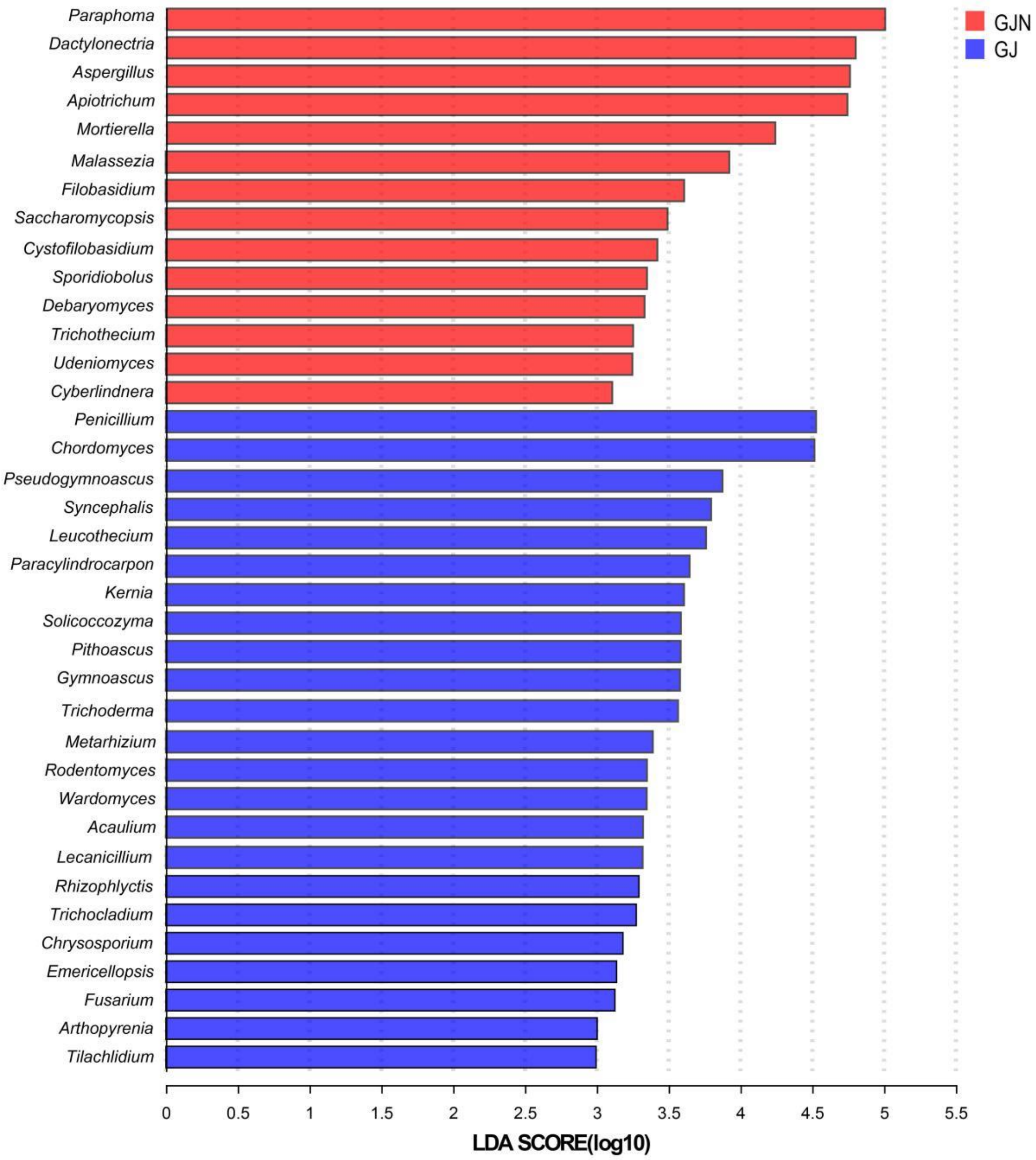

Figure S9. The LEfSe analysis the significantly enriched fungal community between GJN group and GJ group. Fungal genera with the LDA score of more than 2 are shown.

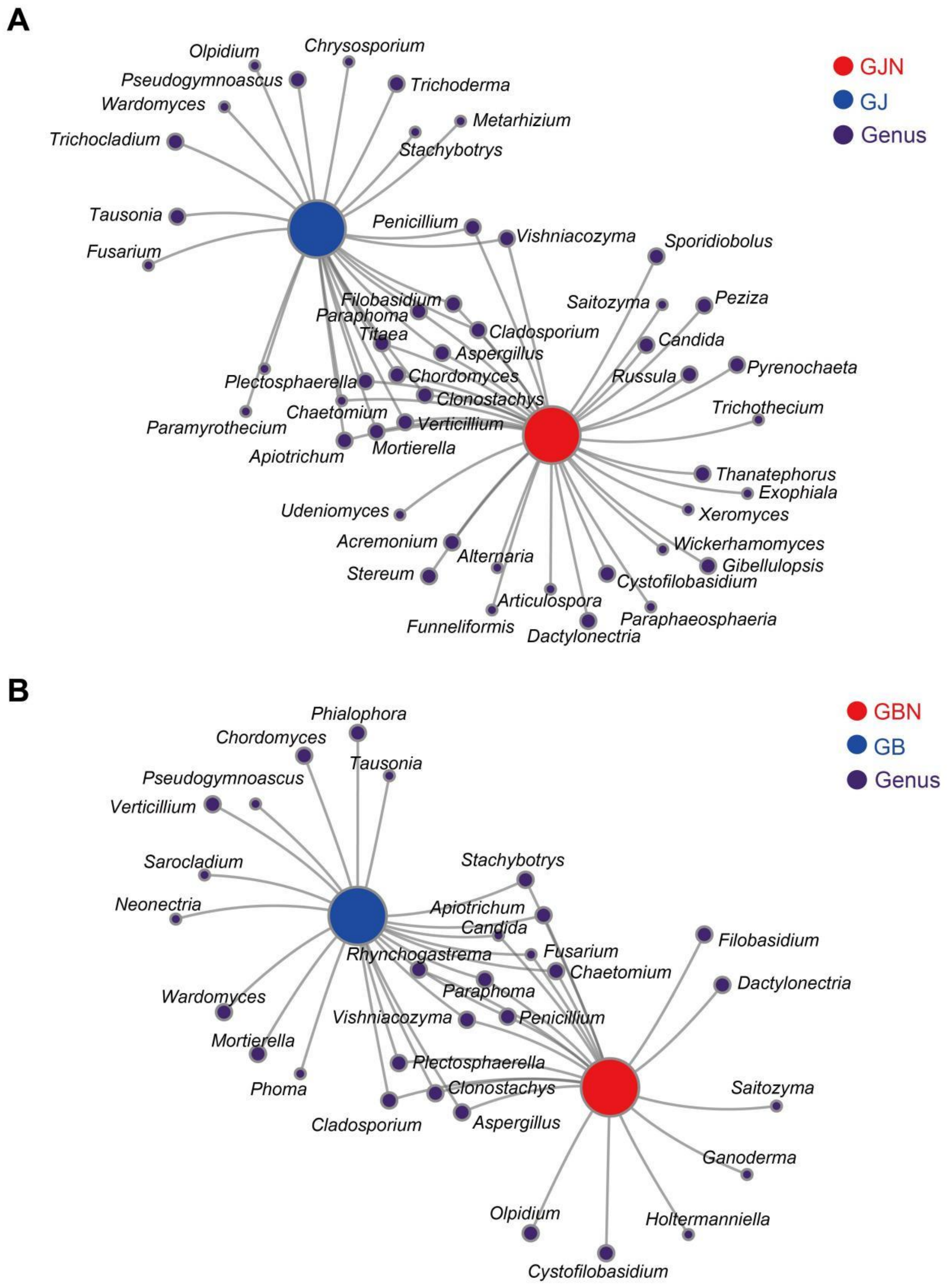

Figure S10. The network analysis the common and unique genera between GJN and GJ (A), and GBN and GB (B).
